# Risk Factors for Ventriculoperitoneal Shunt Infection: A Systematic Review and Meta-Analysis

**DOI:** 10.3390/brainsci15101055

**Published:** 2025-09-28

**Authors:** Francesco Signorelli, Matteo Palermo, Francesco Onorati, Fabio Zeoli, Marina Romozzi, Giammaria Marziali, Carmelo Lucio Sturiale, Gianluca Trevisi, Massimiliano Visocchi

**Affiliations:** 1 Department of Neurosurgery, Fondazione Policlinico Universitario Agostino Gemelli IRCCS, Università Cattolica del Sacro Cuore, 00168 Rome, Italy; 2 Department of Neuroscience, Fondazione Policlinico Universitario Agostino Gemelli IRCCS, Università Cattolica del Sacro Cuore, 00168 Rome, Italy; 3 Department of Radiology, Fondazione Policlinico Universitario Agostino Gemelli IRCCS, Università Cattolica del Sacro Cuore, 00168 Rome, Italy

**Keywords:** hydrocephalus, ventriculoperitoneal shunt, infection

## Abstract

**Background:** Ventriculoperitoneal shunting (VPS) is the mainstay of treatment for most forms of hydrocephalus; VPS infection (VPSI) is a leading cause of shunt-related morbidity and mortality. A meta-analysis of the existing literature on risk factors for VPSI is currently lacking. Herein, the authors performed a systematic review and meta-analysis to evaluate the role of different clinical factors in the development of VPSI. **Methods:** A systematic search in the PubMed, Scopus, and Cochrane databases was performed to identify studies comparing patients developing VPSI to controls. The following data were extracted where available: number of patients who developed VPSI vs. number of patients with a regular course, demographics (gender, age at VPS insertion, age > 18 years), average length of hospital stay before shunt implant (days), aetiology of hydrocephalus (tumour-associated hydrocephalus; post-haemorrhagic hydrocephalus; congenital hydrocephalus; spinal dysraphism-associated hydrocephalus; post-infectious hydrocephalus; post-traumatic hydrocephalus; post-craniotomy hydrocephalus), and hydrocephalus type (obstructive hydrocephalus, communicating hydrocephalus, normal-pressure hydrocephalus—NPH). **Results:** Five studies including 2333 patients (225 with VPS infection) were analysed. Tumour-related hydrocephalus was linked to a lower infection risk (OR 0.418; *p* < 0.001), while congenital hydrocephalus (OR 2.502; *p* < 0.001) and spinal dysraphism (OR 2.359; *p* < 0.001) increased the risk. **Conclusions:** VPSI represents a serious complication after shunt surgery. Our meta-analysis identifies three key factors influencing the risk of VPSI. VPS-centred, large multicentre prospective studies are needed to possibly confirm the role of the factors we identified and to identify additional ones, enabling earlier detection of VPSI and allowing for better patient care.

## 1. Introduction

Cerebrospinal fluid (CSF) shunts are widely used in the treatment of hydrocephalus to divert CSF to another body cavity, where it can be reabsorbed. Among the available techniques, ventriculoperitoneal shunting (VPS) represents the mainstay of treatment and remains one of the most frequently performed procedures in neurosurgical practice [1]. However, VPS infection (VPSI) is a leading cause of shunt malfunction [2], with reported infection rates ranging from 5.6% to 12.9% in patients undergoing VPS placement [1,3]. Shunt infection can lead to ventriculitis and increased mortality [4]. For patients with a previous shunt infection, re-infection is quite common [5]. Both in adult and paediatric populations, shunt infection usually occurs within a few months after shunt surgery, and is associated with a substantial risk of morbidity, including increased risk of seizures and decreased cognitive performance [6,7,8].

Moreover, despite advancements in shunt system, valve design, and sterilization techniques, prevention of shunt-associated complications has failed to demonstrate significant progress over the past few decades [9]. VPSI is still a common complication entailing hospital readmission and additional treatment, thus increasing VPS-associated morbidity, mortality, and healthcare costs [10]. The type of valve used may also influence the risk of shunt infection. Programmable valves offer the advantage of adjustability but usually require more handling during surgery and sometimes later adjustments, which could increase opportunities for contamination. Fixed-pressure valves often need replacement if the pressure setting is no longer adequate, and each revision carries an added risk of infection [10]. More recently, antibiotic-impregnated valves and catheters have been developed, and several studies suggest they can reduce the rate of early postoperative infections, although their benefit may diminish over time. These aspects highlight that valve choice is not only a technical decision, but may also shape the patient’s overall infection risk [10].

The initial therapeutic approach often consists of removal of the infected system, placement of a temporary external CSF drain, and a prolonged course of targeted antibiotic therapy, followed by the insertion of a new shunt system, which often requires prolonged hospitalization, lasting up to two or three weeks [11,12]. Indeed, VPSI accounts for about 21.1% of shunt revision procedures [13,14]. A recent meta-analysis performed by our group found a higher risk of infection with VPS comparing with LPS, probably due to a shorter hardware placed in LPS (only 1.5% of LPS cases versus 5% with VPS) [15].

Several studies have attempted to identify risk factors associated with VPSI, most commonly focusing on patient age, hydrocephalus type and etiology, duration of the shunt procedure [8,16], presence of a pre-existing shunt system [17], and the occurrence of postoperative CSF leakage [18]. However, a comprehensive meta-analysis synthesizing the available evidence is currently lacking. Herein, the authors performed a systematic review and meta-analysis of the existing literature to evaluate the role of aetiology and clinical factors in the development of VPSI.

## 2. Methods

A systematic search in the PubMed, Scopus, and Cochrane databases was performed according to the Preferred Reporting Items for Systematic Reviews and Meta-Analysis (PRISMA) guidelines [19]. The research was launched on 31 March 2025, using the following: “((VPS OR ventriculoperitoneal OR ventriculo-peritoneal) AND (shunt))”. No restrictions on the date of publication were made. Studies published in languages other than English, conference abstracts, case reports, and case series (*n* < 10), series reporting mixed shunt types in addition to VPS (namely, ventriculo-atrial, lumbo-peritoneal, ventriculo-peritoneal) that did not allow for the differentiation of data and outcomes specifically attributable to “VPS”, and purely paediatric series (mean age < 18 years) were excluded from full-text review.

Two authors (M.P. and F.O.) independently screened the abstracts for eligibility. Any discordance was solved by consensus with a third author (F.S.). A systematic abstract screening of the references (forward search) was also performed to identify additional relevant studies.

**Figure 1:**
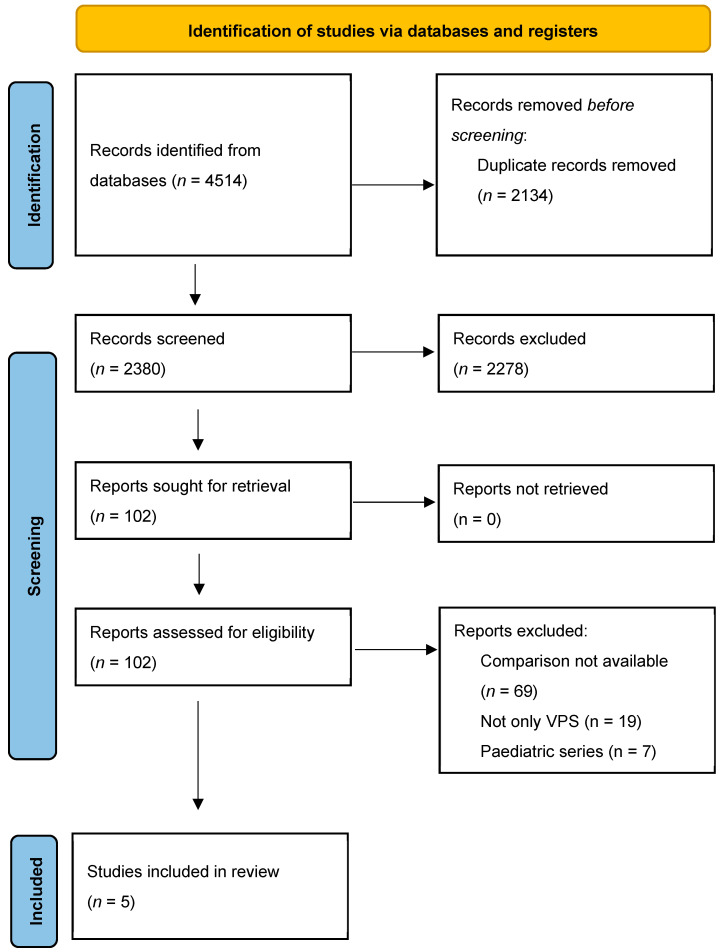
PRISMA flow diagram for the systematic review and meta-analysis detailing the database searches, the number of abstracts screened, and the full texts retrieved.

A PRISMA flowchart depicting the selection and screening process is provided in Figure 1.

Although a standard definition of VPSI is lacking in the literature, we defined VPSI as the presence of at least one of the following criteria: (1) isolation of the organism from 1 or more CSF cultures or (2) clinical findings suggestive of infection (e.g., temperature > 38 °C, headache, neck stiffness, cranial nerve signs, or irritability without another recognized cause) and typical CSF findings (high CSF leukocyte count, high CSF total protein level, and hypoglycorrhachia, as defined by the individual laboratory parameters).

### 2.1. Data Extraction

For each included study for the meta-analysis we collected: number of patients who developed VPSI vs. number of patients with a regular course, aetiology of hydrocephalus (tumour-associated hydrocephalus; congenital hydrocephalus; spinal dysraphism-associated hydrocephalus). We also attempted to collect data on additional variables, including patient demographics (gender; age at VPS insertion; average length of hospital stay prior to shunt placement), as well as other aetiologies (e.g., post-haemorrhagic, post-infectious, post-traumatic, and post-craniotomy hydrocephalus) and the presence of normal-pressure hydrocephalus (NPH), obstructive or communicating hydrocephalous. However, data on these parameters were insufficient or inconsistently reported across studies, precluding their inclusion in the meta-analysis. We did not impose strict selection criteria on the potential risk factors; rather, we reported and analysed those consistently available across the literature and suitable for meta-analysis.

### 2.2. Risk of Bias

**Figure 2:**
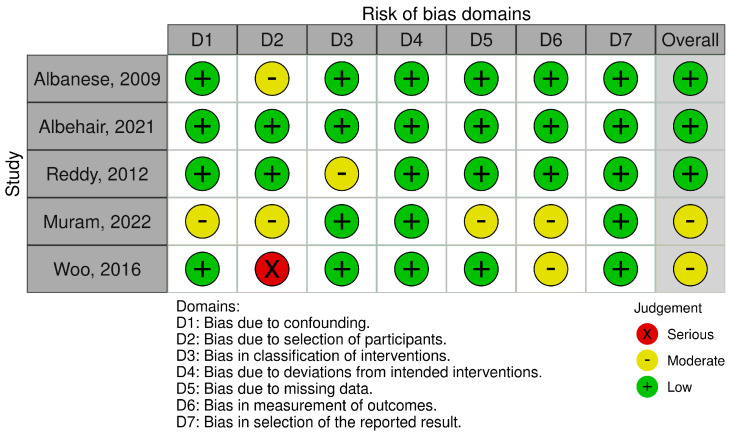
Risk of bias—quality assessment of the included studies [20,21,22,23,24].

The ROBINS-I V2 (Risk Of Bias In Non-randomized Studies of Interventions, Version 2) assessment tool along with the robins application (https://mcguinlu.shinyapps.io/robvis/ (accessed on 2 April 2025)) were used to evaluate study quality through visual representations (Figure 2).

### 2.3. Statistical Analysis

For studies reporting sample size along with median and range or interquartile range, but lacking mean and standard deviation, these latter values were estimated using the methods proposed by Luo et al. and Wan et al. [25,26]. Statistical analyses were performed using OpenMeta (V 3.1) [Analyst] (Brown University, Providence, RI, USA) applying the random effect model. Heterogeneity was tested using the chi-square test and quantified by calculating the I^2^ statistic, in which *p* < 0.05 and I^2^ > 50% were considered statistically significant. For the pooled effects, odds ratios (OR) were calculated for dichotomous variables. Publication bias was tested using the funnel plot.

## 3. Results

The search of the literature yielded a total of 4514 results. Via title and abstract screening, 102 studies were found to be potentially relevant to the present study and thus included for full-text review (Figure 1). Upon full-text review, five articles met the inclusion criteria (Table 1), refs. [20,21,22,23,27] including 2333 patients, of which 225 received a diagnosis of VPSI, with an overall infection rate ranging between 5.1% and 38.8%.

**Figure 3:**
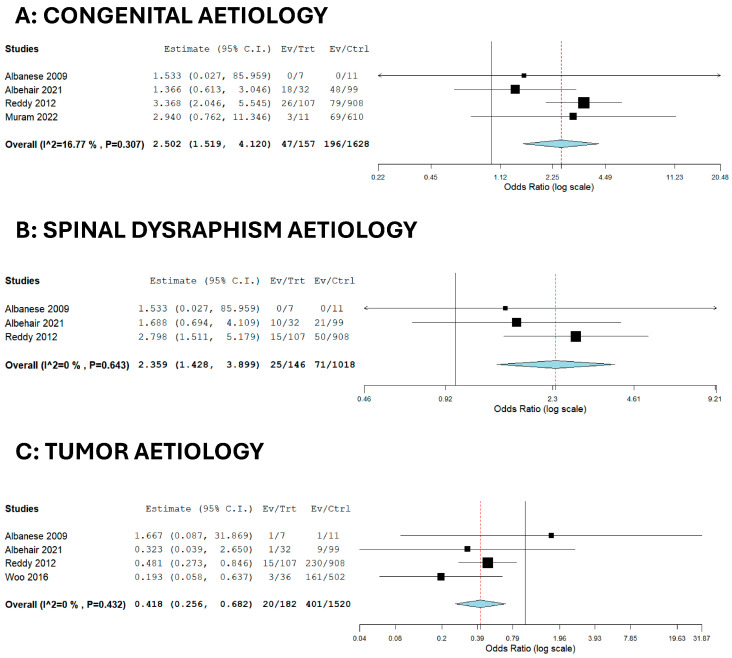
Forest plots for significant variables [20,21,22,23,24].

The following factors were associated with a higher risk of infection after VPS placement for hydrocephalus: congenital aetiology (29.9% of infected patients vs. 12.0% of noninfected patients; OR = 2.502; Table 2, Figure 3A) and spinal dysraphism-associated hydrocephalus (17.1% of infected patients vs. 6.97% of noninfected patients; OR = 2.359; Table 2, Figure 3B). The only factor associated with a lower risk of infection was tumour-associated hydrocephalus (10.9% of infected patients vs. 26.38% of noninfected patients; OR = 0.418; Table 2, Figure 3C). The funnel plots were largely symmetrical for all of the studied factors.

The ROBINS-I assessment indicated that the most frequent sources of bias were confounding, patient selection, and outcome measurement. Several studies lacked adequate adjustment for clinical covariates, enrolled highly selected populations, or reported outcomes without blinded assessment. Incomplete follow-up further contributed to potential bias.

## 4. Discussion

Our meta-analysis provides evidence for higher infection rates in congenital and spinal dysraphism-associated hydrocephalus (Figure 3A,B), while tumour-related cases showed a lower risk (Figure 3C). Several factors may explain the tumour-related lower risk finding (OR = 0.418): these patients are usually older, undergo surgery in more controlled settings, and often require fewer prior neurosurgical procedures, all of which may reduce exposure to infection [20]. In malignant tumours, the limited survival time may also shorten the window in which a shunt infection could develop. Still, some subgroups, such as glioblastoma patients who develop communicating hydrocephalus after surgery and radiotherapy, remain at risk and warrant close follow-up [20].

On the other hand, the elevated infection risk observed in congenital (OR = 2.502) and spinal dysraphism-associated hydrocephalus (OR = 2.359) may be explained by several factors. These patients often undergo multiple neurosurgical procedures from an early age, which increases cumulative exposure to potential contamination. They are also typically shunt-dependent for longer periods, further amplifying the lifetime risk of infection. In spina bifida, skin fragility and impaired wound healing may compromise the surgical field, while complex comorbidities and frequent hospitalizations can promote colonization by multidrug-resistant organisms. Together, these elements provide a plausible causal basis for the higher infection rates consistently observed in these groups.

Recently, Alkosha et al. [28] proposed a protective surgical protocol aiming at reducing shunt infection. The authors identified several key intraoperative measures that were significantly associated with a lower risk of infection. These included double-gloving throughout the procedure to minimize bacterial contamination, intraoperative irrigation of both the surgical wound and the shunt components with vancomycin-infused saline solution to provide localized antimicrobial coverage, and the use of adhesive incision drapes to create a barrier between the skin flora and the surgical field. Their findings suggest that adopting a multimodal infection prevention strategy may provide measurable benefits in decreasing VPSI rates, particularly in high-risk patient populations.

However, while conservative management with systemic antibiotic therapy is possible, it yields an unsatisfactory cure rate. As a result, a combination of targeted antibiotic therapy and complete hardware removal is often required to achieve definitive control of the infection [13]: in the study by Shurtleff et al., complete shunt removal in patients with *S. epidermidis* shunt infections yielded a 100% cure rate, while systemic antibiotics alone were associated with only a 9% cure rate [29]. As such, the identification of risk factors for the development of VPSI is paramount to guiding preventive strategies and improving patient outcomes.

Additionally, one of the studies included in this meta-analysis (Reddy et al.) [22] similarly described a higher incidence of infection among patients with spinal dysraphism-associated/congenital hydrocephalus compared to our pooled data. While this finding may partly reflect the younger age of the cohort, which is generally associated with a higher risk of infection, it may also be attributed to other contributing factors [30,31,32]. These include the higher frequency of prior neurosurgical interventions, longer cumulative shunt dependency, and potential anatomical or skin integrity challenges associated with congenital malformations. Furthermore, patients with spina bifida often have complex medical comorbidities and a higher risk of colonization with multidrug-resistant organisms, which may further elevate infection risk. Notably, our findings are in line with those of Woo et al. [23] (included in our review), whose large multicentre study including infants, paediatric patients, and adults did not find age to be an independent risk factor for shunt infection, suggesting that etiology is a more critical determinant than age alone. In their study, Woo et al. [23] defined shunt infection according to the following criteria: (1) isolation of a pathogenic microorganism from cerebrospinal fluid (CSF) or shunt hardware in the presence of compatible signs and symptoms of central nervous system infection or shunt malfunction; (2) surgical site infection requiring shunt reinsertion, as defined by the National Nosocomial Infection Surveillance System; or (3) the formation of an intraperitoneal pseudocyst. These broad and clinically meaningful criteria reinforce the relevance of correlating microbiological, clinical, and surgical findings in diagnosing VPSI.

In the literature, spinal dysraphism-associated hydrocephalus treated with VPS has consistently been associated with a variably high incidence of VPSI [33,34]. One proposed contributing factor is the timing of myelomeningocele repair surgery. Several studies have reported that simultaneous repair of myelomeningocele and VPS placement is associated with a greater VPSI rate [35,36]. Indeed, myelomeningoceles are generally regarded as clean-contaminated or contaminated wounds, thus the insertion of a foreign body (shunt) into an already contaminated field during the repair surgery may increase the incidence of shunt infections. Consequentially, some authors advocate for delaying VPS insertion after myelomeningocele repair surgery. This hypothesis could not be explored in our meta-analysis because a subgroup analysis based on the timing of myelomeningocele repair and VPS insertion was not feasible.

Our meta-analysis found that tumour aetiology was associated with a lower rate of VPSI. Similarly, Reddy et al. defined tumour-associated hydrocephalus as a low-risk condition for shunt infection, mainly due to the older age of the affected cohort. One possible explanation is that tumour-associated hydrocephalus in adults often results from rapidly progressing neoplasms compressing on the ventricular system or from adjuvant radiotherapy, particularly in cases like glioblastoma [37,38]. However, the inherently poor prognosis and limited survival may recede the window of time in which VPSI could develop, thus possibly explaining our decreased odds.

Furthermore, the study by Reddy et al. also showed further insights on the VPSI rates between communicating and obstructive hydrocephalus. Specifically, obstructive hydrocephalus was associated with an increased risk of VPSI (OR: 1.751), while communicating hydrocephalus showed a protective association (OR: 0.505). Although both findings reached statistical significance, the effect sizes were modest, and the low to moderate heterogeneity (I^2^ = 0% and 18%, respectively) suggests relatively consistent results across the included studies.

These findings may be explained by the underlying pathophysiology as patients with obstructive hydrocephalus often present with more acute or localized blockages, sometimes secondary to mass lesions or aqueductal stenosis, may present with elevated intraventricular pressures and more urgent indications for shunting, potentially increasing surgical complexity and infection risk. In contrast, communicating hydrocephalus, especially NPH or post-meningitis hydrocephalous, allow for elective and well-controlled surgical planning, thus reducing the infection risk. This is because patients are often clinically stable at the time of surgery, with lower CSF pressures, facilitating cleaner operative fields and technically simpler procedures.

Indeed, several authors reported an increased risk in cases where shunt surgery is preceded by external ventricular drainage (EVD) [39,40,41], as in post-haemorrhagic hydrocephalus. Early conversion to an internal CSF shunt seems to decrease the risk of drain related infection [42,43].

In the available literature, other factors that may affect VPSI development include the use of antibiotic-impregnated shunts [20]; previous shunt infection from certain bacterial species prone to drug resistance, such as *S. aureus* [24,44]; and the occurrence of postoperative CSF leaks [33]. However, such factors were not meta-analysed because of the heterogeneous data of the studies included.

Beyond patient-related characteristics, several surgical and hospital-related variables have also been consistently reported as important determinants of VPSI. Factors such as prolonged operative duration, surgery performed during off-peak hours, limited surgeon experience, valve design, and the use of antibiotic-impregnated shunts have all been shown to influence infection risk in previous series. Because of heterogeneous reporting and the inclusion of mixed shunt populations, these variables could not be quantitatively assessed in our meta-analysis. Nevertheless, their potential impact should not be underestimated, and our results should be interpreted alongside these well-recognized contributors. Future prospective, VPS-focused multicentric studies should integrate both clinical and surgical determinants to provide a more comprehensive framework for infection prevention and patient care.

## 5. Limitations

Our meta-analysis aimed to find risk factors and preventive factors for the development of VPSI in adults, so as to permit the early identification of patients most susceptible to infection for a closer monitoring. Because of the heterogeneous data of the study we included, we only meta-analysed clinical variables. Surgical variables (i.e., operation length, type of valve, surgery performed in the off-peak hours, etc.) could not be included, although their role in VPSI development has been previously described. However, several studies focusing on surgical variables were excluded during the full-text screening process because they did not meet our inclusion criteria (i.e., they included patients with VPS and other forms of shunts, or they set a different endpoint, such as shunt revision rather than infection). While their role in VPSI development is well established, a quantitative or even qualitative synthesis was not feasible within the scope of our analysis. At the same time, the low number of studies included in our meta-analysis entails incompletely reported individual factors and large confidence intervals. This highlights the necessity of VPS-centred, large multicentre prospective studies to identify other risk factors, enabling an earlier detection of VPSI and allowing for better patient care.

## 6. Conclusions

VPSI remains a major complication despite advances in technique and device design. Our meta-analysis found higher infection rates in congenital and spinal dysraphism-associated hydrocephalus, while tumour-related cases showed a lower risk. Age was not associated with VPSI. Other potential risk factors, such as prior external drainage, antibiotic-impregnated shunts, and timing of myelomeningocele repair, could not be fully assessed due to data heterogeneity. Further research is needed to identify effective preventive strategies.

## Figures and Tables

**Table 1:** Studies included in the meta-analysis.

Author	Year	Type of Study	N of Patients (Non-Infected/Infected)
Albanese et al. [20]	2009	Retrospective	28 (11/7)
Reddy et al. [22]	2012	Retrospective	1015 (908/107)
Woo et al. [23]	2016	Retrospective	538 (502/36)
Albehair et al. [21]	2021	Retrospective	131 (99/32)
Muram et al. [24]	2022	Retrospective	621 (610/11)

**Table 2:** Meta-analysis of possible factors associated with infection after a DVP placement for hydrocephalus.

Factor	OR	95% CI	*p*-Value	I^2^
Tumor	0.418	0.256–0.682	<0.001	0
Congenital	2.502	1.519–4.120	<0.001	16.77
Spinal dysraphism	2.359	1.428–3.899	<0.001	0

OR: odds ratio; CI: confidence interval.

## Data Availability

No new data were created or analyzed in this study. Data sharing is not applicable to this article.

## Abbreviations

CSF	Cerebrospinal fluid
PRISMA	Preferred Reporting Items for Systematic Reviews and Meta-Analysis
NPH	Normal pressure hydrocephalus
VPS	Ventriculoperitoneal shunt
VPSI	Ventriculoperitoneal shunt infection
